# The Potential of Gut Microbiota Metabolic Capability to Detect Drug Response in Rheumatoid Arthritis Patients

**DOI:** 10.3389/fmicb.2022.839015

**Published:** 2022-04-08

**Authors:** Maozhen Han, Na Zhang, Yujie Mao, Bingbing Huang, Mengfei Ren, Zhangjie Peng, Zipeng Bai, Long Chen, Yan Liu, Shanshan Wang, Shenghai Huang, Zhixiang Cheng

**Affiliations:** ^1^School of Life Sciences, Anhui Medical University, Hefei, China; ^2^Department of Blood Transfusion, The Fourth Affiliated Hospital of Anhui Medical University, Hefei, China; ^3^Department of Maternal, Child, and Adolescent Health, School of Public Health, Anhui Medical University, Hefei, China; ^4^Department of Microbiology, School of Basic Medical Sciences, Anhui Medical University, Hefei, China

**Keywords:** gut microbiota, rheumatoid arthritis, methotrexate, drug metabolism, clinical medication

## Abstract

Gut microbiota plays an essential role in the development of rheumatoid arthritis (RA) and affects drug responses. However, the underlying mechanism remains elusive and urgent to elucidate to explore the pathology and clinical treatment of RA. Therefore, we selected methotrexate (MTX) as an example of RA drugs to explore the interactions between the gut microbiota and drug responses and obtain an in-depth understanding of their correlation from the perspective of the metabolic capability of gut microbiota on drug metabolism. We identified 2,654 proteins and the corresponding genes involved in MTX metabolism and then profiled their abundances in the gut microbiome datasets of four cohorts. We found that the gut microbiota harbored various genes involved in MTX metabolism in healthy individuals and RA patients. Interestingly, the number of genes involved in MTX metabolism was not significantly different between response (R) and non-response (NR) groups to MTX, but the gene composition in the microbial communities significantly differed between these two groups. Particularly, several models were built based on clinical information, as well as data on the gene, taxonomical, and functional biomarkers by using the random forest algorithm and then validated. Our findings provide bases for clinical management not only of RA but also other gut microbiome–related diseases. First, it suggests that the potential metabolic capability of gut microbiota on drug metabolism is important because they affect drug efficiency; as such, clinical treatment strategies should incorporate the gene compositions of gut microbial communities, in particular genes involved in drug metabolism. Second, a suitable model can be developed to determine hosts’ responses to drugs before clinical treatment.

## Introduction

The human microbial community consists of more than 100 trillion microbes, most of which are unculturable and mainly colonize the intestinal tract (Manzo and Bhatt, 2015). These microbes evolved with the hosts and become an inalienable part of the hosts (Glasner, 2017). Increasing lines of evidence have demonstrated that the human gut microbiota is associated with human health and plays an essential role in maintaining the homeostasis and health of hosts. For instance, numerous studies have proven that human gut microbiota is involved in the metabolism and immune system development of hosts; it also contributes to host physiology and is associated with the development of inflammatory diseases, such as rheumatoid arthritis (RA) (Manasson et al., 2020), juvenile idiopathic arthritis (Tejesvi et al., 2016), systemic lupus erythematosus (Chen et al., 2021), Crohn disease, ulcerative colitis, and irritable bowel syndrome (Wu and Wu, 2012; Carding et al., 2015), and other diseases, such as hypertension (Li et al., 2017), cholestatic liver disease (Isaacs-Ten et al., 2020), and cancers (Amy et al., 2020; Slowicka et al., 2020). However, although many studies investigated the pathology of diseases and established the correlation between the dynamic changes of human gut microbiota and diseases (Bäckhed et al., 2015; Ghaisas et al., 2016; Gaulke and Sharpton, 2018), the correlation analysis was conducted only in a certain cohort and the causality of the disease remains elusive and urgent to elucidate using a large of metagenome datasets. Besides, databases of reference genes and proteins that are associated with a certain disease, particularly those involved in drug metabolism, are needed for rapid analysis of metagenome datasets.

Rheumatoid arthritis is an autoimmune disease that has chronic inflammation symptoms and disorders. In general, RA can cause joint pain, swelling, and stiffness, as well as damages throughout the body, not only on the skin, eyes, lungs, and heart (Majithia and Geraci, 2007; Bullock et al., 2018). To date, although the pathology of RA remains not fully understood, many studies have reported that the occurrence and development of RA are associated with genetic and environmental factors (Majithia and Geraci, 2007; Aletaha and Smolen, 2018), including oral and gut microbiota (Maeda and Takeda, 2017), smoking, age, body mass index, and so on (Rydell et al., 2018). The experiment results in germ-free mice revealed that the gut microbiota can shape the intestinal immune system and has a strong positive association with the diseases of the immune system (Round and Mazmanian, 2009; Torres et al., 2020), including RA. The dysbiosis of human gut microbial communities can cause disorders of the immune system in RA patients; numerous studies have also reported the dynamic changes of oral and gut microbiome between RA patients and healthy controls (Zhang et al., 2015; Horta-Baas et al., 2017; Brown et al., 2020). Among gut microorganisms, *Prevotella copri* was significantly dominant in RA patients and strongly correlated with the absence of human leukocyte antigen–DRB1 (Scher et al., 2013). Besides, the hidden links in the gut–joint axis for RA have been demonstrated, and the ascorbate degradation of gut microbes contributes to the development of RA has been proposed (Zhao et al., 2021). This finding provides new avenues for the prevention and treatment of RA (Cassotta et al., 2021). Together, these results suggest that the gut microbiota plays an important role in the occurrence and development of RA; hence, the clinical treatment of RA should consider gut microbiome approaches.

At present, although the pathology of RA remains unknown, many drugs have been developed and applied to its clinical treatment; these drugs include methotrexate (MTX), ibuprofen, naproxen, prednisolone, sulfasalazine, leflunomide, and other disease-modifying antirheumatic drugs (DMARDs) (Aletaha and Smolen, 2018). MTX, as one of the DMARDs, has been recommended as the first-line drug for the clinical treatment of RA among several treatment guidelines (Bello et al., 2017). However, MTX is insufficient to control the symptoms of patients regardless of the mode of delivery, and up to 50% of patients do not obtain a clinically effective outcome (Bello et al., 2017; Yamakawa et al., 2021). The phenomenon of different responses for the same drug in RA patients could be due to variations in their gut microbiome (Zhang et al., 2015; Kishikawa et al., 2020; Artacho et al., 2021). However, although several studies focused on responses to drugs, and the dynamic changes of the gut microbiome have been conducted, the underlying mechanism remains elusive, and the potential metabolic capability of the gut microbiota on drug metabolism is unclear.

Hence, together with several recent studies (Zhang et al., 2015; Kishikawa et al., 2020; Artacho et al., 2021), there is no doubt that the gut microbiota plays an essential role in the occurrence and development of RA, and the drug responses in RA patients depend on the gut microbiome of the host. However, the detailed mechanism, particularly the potential metabolic capability of the gut microbiome, remains elusive and urgent to elucidate to explore RA pathology and develop a microbiome-based approach for detecting hosts’ responses to drugs before clinical treatment of RA. In this present work, we collected the gut metagenome datasets related to RA and the corresponding clinical information of the host. Only the BioProject PRJNA682730 has the information on drug response. Considering the clinical application of MTX, we selected it as an example of RA drugs to explore the relationship between gut microbiota and drug response. Specifically, we collected the gut metagenome datasets of healthy individuals and RA patients from four cohorts. We cross-assembled the gut metagenome datasets of the training dataset of PRJNA682730, identified the proteins and genes involved in MTX metabolism, calculated their abundance and presence in the gut microbiome datasets of the four cohorts, constructed several machine models with the random forest algorithm, and verified the models in the testing data of PRJNA682730. Our findings suggest that the potential metabolic capability of gut microbiota evaluated by the composition of the genes involved in drug metabolism affected the efficiency of drugs and can be applied to detect the drug response of RA patients, which elicits effects on clinical management of gut microbiome-related diseases.

## Materials and Methods

### Construction of Protein Databases Involved in the Metabolism of Clinical Drugs for Rheumatoid Arthritis

**FIGURE 1:**
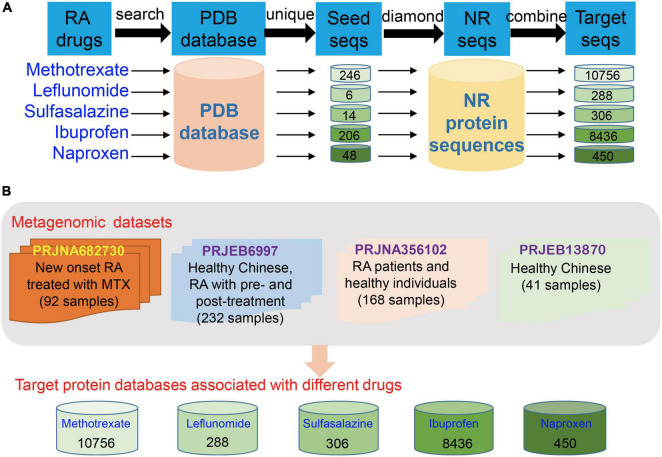
Construction of protein databases for clinical drugs in rheumatoid arthritis (RA) treatment and the human gut metagenome datasets used in the present study. **(A)** Construction of databases for detection of proteins and the corresponding genes involved in drug metabolism in the gut microbial communities of RA patients. **(B)** Gut metagenome datasets used in this study were collected from four cohorts, namely, BioProject PRJNA682730, PRJEB6997, PRJNA356102, and PRJEB13870 (only the dataset of healthy individuals was used).

Several clinical drugs, including MTX, leflunomide, sulfasalazine, ibuprofen, naproxen, and other DMARDs (Aletaha and Smolen, 2018), have been widely applied to the clinical treatment of RA. Although MTX, a famous clinical DMARD, has been recommended as the first-line drug for the clinical treatment of RA among several treatment guidelines, more than 50% of RA patients who received it do not achieve a clinically effective outcome. The reason and detailed mechanism remain unclear but may be dependent on the gut microbiome. Hence, we selected MTX, which is a typical and preferred drug in the clinical treatment of RA, to obtain an in-depth understanding of the interactions between the gut microbiota and drug response and reveal the potential metabolic capability of gut microbiota on drug metabolism in RA treatment. Specifically, we collected proteins that used MTX as a ligand and were involved in its metabolism from the PDB database (length > 50 amino acid residues). And then we extracted the protein sequences from the NR databases and evaluated the protein database by using Diamond (similarity > 90% and query coverage > 90%; Figure 1A). Finally, we collected 10,756 protein sequences and used them to construct the database for downstream analysis of MTX (Figure 1A). Protein databases for other drugs were also built (Figure 1). These databases can be obtained and downloaded from Github.^1^

### Study Design and Group Information of Metagenome Datasets Collected From Four Cohorts

In this present study, we collected the gut metagenome datasets from four human cohorts to explore the interactions between the gut microbiota and drug response of the host from the perspective of the potential metabolic capability of gut microbiota on drug metabolism. The cohorts are as follows: PRJNZ682730 (92 fecal samples, divided into three groups, namely, training data [49 samples of RA patients treated with MTX, including 20 samples with response to MTX and 29 samples without response to MTX, Supplementary Table 1], testing data [21 samples of RA patients with the treatment of MTX, including nine samples with response to MTX and 12 samples with non-response to MTX], and other DMARD group [22 samples of RA patients with the treatment of other drugs, five samples with response to the other drug and 17 samples with non-response to the other drugs]; information of these samples whether response [R] or non-response [NR] to MTX was recorded) (Artacho et al., 2021); PRJEB6997 (healthy Chinese and Chinese RA patients who underwent pretreatment and posttreatment, 232 fecal samples, the group information is unclear) (Zhang et al., 2015); PRJNA356102 (RA patients and healthy individuals, 168 fecal samples); and the healthy individuals of PRJEB13870 (41 fecal samples, these samples were used as control; Figure 1B) (Li et al., 2017). Thus, a total of 533 human gut metagenome datasets were collected to profile taxonomical and functional compositions, and the abundances of potential genes involved in MTX metabolism of gut microbial communities. Particularly, the gut metagenome datasets of the training and testing data of the BioProject PRJNZ682730 were used to investigate differences between the R and NR groups, construct, and verify the models, respectively.

### Metagenome Dataset Processing

Quality control was first conducted on the human gut metagenome datasets collected from the four cohorts against the human genome (version hg38) by using bowtie2 to remove the contaminations of humans. Then, the quality of the metagenome datasets was controlled with Trimmomatic (version 0.32) (Bolger et al., 2014) with the following parameters: TruSeq3-PE.fa: 2:30:10, leading: 3, trailing: 3, sliding window: 5:20, and min length: 25, to remove adaptors and filter the low-quality sequences. The high-quality sequences were used for downstream analysis.

### Genome Assembly, Gene Prediction, and Functional Annotation

To evaluate the potential metabolic capability of gut microbiota on MTX metabolism across the four cohorts, we selected the metagenome datasets of the training data in the BioProject PRJNZ682730 and cross-assembled the datasets of 49 samples (Figure 1B and Supplementary Table 1). *De novo* metagenome assembly was performed with MEGAHIT (version 1.2.9) (Li et al., 2016), with option –meta-large and with a *k*-mer list of 27, 37, 47, 57, 67, 77, 87, 97, 107, 117, and 127. Contigs larger than 1,000 bp were kept for further analysis. Prodigal (version 2.6.2) in the “Meta” model was applied to detect the complete open reading frames and proteins in the assembled contigs (Hyatt et al., 2010). Diamond was applied to identify the protein involved in MTX metabolism with the following parameters: similarity >30% based on the threshold of homology modeling and query coverage >70% (Kurella and Gali, 2014). Thus, we identified 2,654 proteins that potentially involved in MTX metabolism. Subsequently, we obtained the corresponding genes and the assembled contigs carrying these genes. The abundance of each gene was calculated according to the equation of reads per kilobase per million mapped reads and then transformed to relative abundance. The compositions of genes involved in MTX metabolism were calculated for all samples. The presence of the 2,654 genes was investigated in the gut metagenome datasets of the four cohorts based on the abundance of genes. In addition, the taxonomy of the assembled contigs carrying genes involved in MTX metabolism was annotated with Contig Annotation Tool (CAT)^2^ (von Meijenfeldt et al., 2019). Besides, the functions of genes attached to the assembled contigs were obtained by annotating the corresponding proteins against the NR database with Diamond (*e* value < 1e-5), and the top annotation was selected as the best functional hit for each gene.

### Taxonomical and Functional Compositions of the Metagenome Datasets

To profile the taxonomical and functional compositions of gut microbial communities in the training and testing data of BioProject PRJNZ682730 (Supplementary Table 1), MetaPhlan3 and HuMAnN3 were applied to obtain the taxonomical composition at phylum, class, order, family, genus, and species levels, and functional composition with KEGG level 2 of gut microbial communities with default settings (Beghini et al., 2021), respectively. High-quality paired-end reads were combined into a single document and used as an input for HuMAnN3.

### Biomarker Analysis

Linear discriminant analysis (LDA) effect size (LEfSe, version 1.0) (Segata et al., 2011) was applied to identify taxonomical and functional biomarkers and biomarkers of genes involved in MTX metabolism between the R and NR groups based on their composition in the training data of PRJNZ682730. The *p*-value for the Kruskal–Wallis test among groups was set at 0.05. The thresholds for the logarithmic LDA score for different gene, taxonomical, and functional features were set at 3.0, 2.5, and 3.0, respectively.

### Statistical Analysis

Statistical analysis was conducted on the R platform. Based on the Bray–Curtis distance matrix, the difference in the profiles of genes involved in MTX metabolism between the R and NR groups was estimated. LDA, a supervised learning approach, was applied to maximize the separation of R and NR groups. Particularly, random forest models using 10-fold cross-valuation were built with the “caret” package in R based on the training data and verified on the testing data of PRJNZ682730. The accuracy and area under the curve (AUC) values of the models built using different strategies were calculated and compared (Han et al., 2020a).

## Results

### Occurrence of Genes Involved in Methotrexate Metabolism Among Healthy Individuals and Rheumatoid Arthritis Patients

**FIGURE 2:**
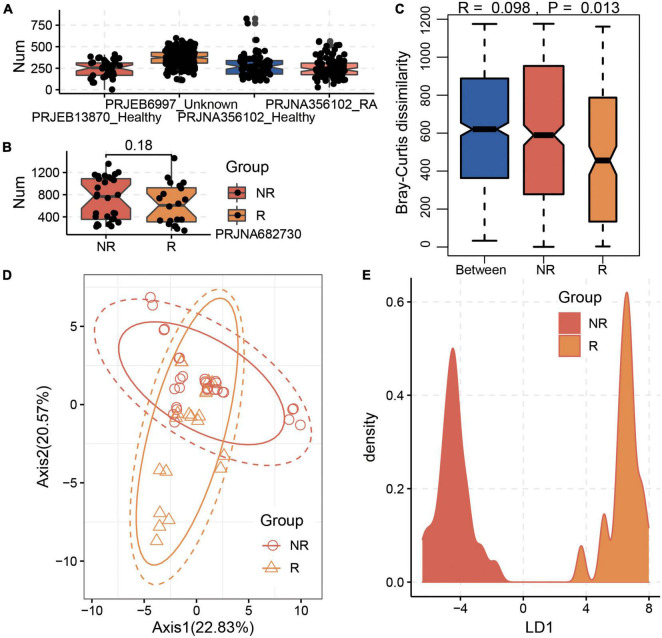
Significant differences in the composition of genes involved in methotrexate (MTX) metabolism between the R and NR groups. **(A)** Gut microbiota harbors various genes involved in MTX metabolism in healthy individuals and RA patients. **(B)** Distribution of genes involved in MTX metabolism in the gut microbiota in the R and NR groups based on the training data of the BioProject PRJNA682730. A significant difference existed between the R and NR groups based on **(C)** Bray–Curtis dissimilarity and **(D)** Principal component analysis (PCA) analysis. **(E)** Linear discriminant analysis (LDA) analysis of the sample distribution showed a distinct separation between the R and NR groups.

After the cross-assembly of the 49 metagenome datasets collected from the training data of the BioProject PRJNZ682730, 552,518 contigs were obtained, and 1,642,925 complete genes/proteins were predicted. Based on the constructed protein database involved in MTX metabolism, we identified 2,654 proteins and the corresponding genes from the gut microbial communities of R and NR samples. We found that the gut microbiota harbored various genes involved in MTX metabolism in healthy individuals and RA patients (Figures 2A,B). Interestingly, we found that although the number of genes involved in MTX metabolism was not significantly different between the R and NR groups (*p* > 0.05, Wilcoxon test; Figure 2B), the compositions of the genes in the microbial communities of the R and NR groups were significantly different (Figures 2C,D; *p* < 0.05, Bray–Curtis dissimilarity). Particularly, the result of LDA showed the distribution of the samples had a distinct separation between the R and NR groups (Figure 2E). This finding indicates that the compositions of genes involved in MTX metabolism of the gut microbial community are differed and suggests that the potential metabolic capability of gut microbiota on MTX metabolism is associated with the host’s response to MTX.

### Biomarkers Analysis and the Construction of Models With Random Forest Algorithm

**FIGURE 3:**
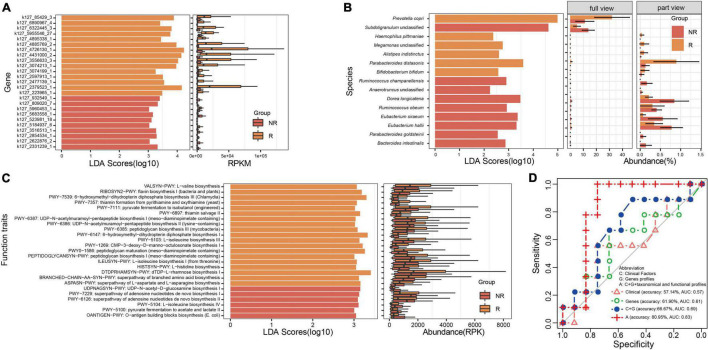
Analysis of biomarkers and the construction of models for distinguishing the samples of the R and NR groups. **(A)** Gene biomarkers involved in methotrexate (MTX) metabolism, **(B)** taxonomical biomarkers at the species level, and **(C)** functional biomarkers identified between the R and NR groups. **(D)** Models were built using the random forest algorithm and verified; the profile of genes involved in MTX metabolism is an important feature that should be used to predict host response to MTX.

To distinguish the samples between the R and NR groups, LEfSe was applied to identify the gene biomarkers involved in MTX metabolism, taxonomical biomarkers, and functional biomarkers between the R and NR groups. Specifically, a total of 25 genes (Figure 3A), including the dihydrofolate reductase gene and methylenetetrahydrofolate dehydrogenase gene, were identified as gene biomarkers. We found that more gene biomarkers were identified in the R group (15 genes) than those in the NR group (10 genes), especially the genes k127_4726130_1 and k127_2379523_1 exhibiting an obvious difference between the two groups (Figure 3A). Moreover, 15 taxonomical species, including *P. copri*, *Parabacteroides distasonis*, *Dorea longicatena*, *Bacteroides intestinalis*, and so on, were identified as taxonomical biomarkers (Figure 3B). In contrast to the distribution of the number of gene biomarkers between the R and NR groups, more taxonomical biomarkers were identified in the NR group (Figure 3B). Besides, 25 functional traits, including the biosynthesis of several kinds of amino acids and the pathway of pyruvate fermentation to acetate and lactate, were identified as functional biomarkers (Figure 3C).

Moreover, to highlight the potential applications of gene biomarkers involved in MTX metabolism, as well as taxonomical and functional biomarkers in clinical treatment, we built several models based on clinical information and gene, taxonomical, and functional biomarkers by using the random forest algorithm and verified them in the testing dataset of PRJNA682730 (Figure 3D). We found that the accuracy of the model built based on gene biomarkers (61.90%, AUC = 0.611) was higher than that based on clinical information (57.14%, AUC = 0.5714). Particularly, the highest accuracy was found in the model built based on clinical information (RF titer), gene biomarkers (k127_4431000_2, k127_5955546_27, k127_932549_1), taxonomical (*P. distasonis*, *Alistipes indistinctus*, *Ruminococcus obeum*, *D. longicatena*, *Anaerotruncus* unclassified, *Subdoligranulum* unclassified), and functional biomarkers (BRANCHED-CHAIN-AA-SYN-PWY: superpathway of branched amino acid biosynthesis, HISTSYN-PWY: L-histidine biosynthesis, OANTIGEN-PWY: *O*-antigen building blocks biosynthesis [*Escherichia coli*], PWY-5103: L-isoleucine biosynthesis III, PWY-6387: UDP-*N*-acetylmuramoyl-pentapeptide biosynthesis I [meso-diaminopimelate containing], PWY-6897: thiamine salvage II, PWY0-1586: peptidoglycan maturation [meso-diaminopimelate containing]; Supplementary Table 1, Figure 3D; 80.95%, AUC = 0.8333); the AUC value is roughly equal to that reported in a previous study (AUC = 0.84, models built with different features) (Artacho et al., 2021). The results suggest that the potential metabolic capability of gut microbiota on MTX metabolism should be considered to determine the drug response of RA patients before the clinical treatment of RA.

### Host-Tracking and Functional Annotations of Genes and Their Physical Distributions in Contigs

**FIGURE 4:**
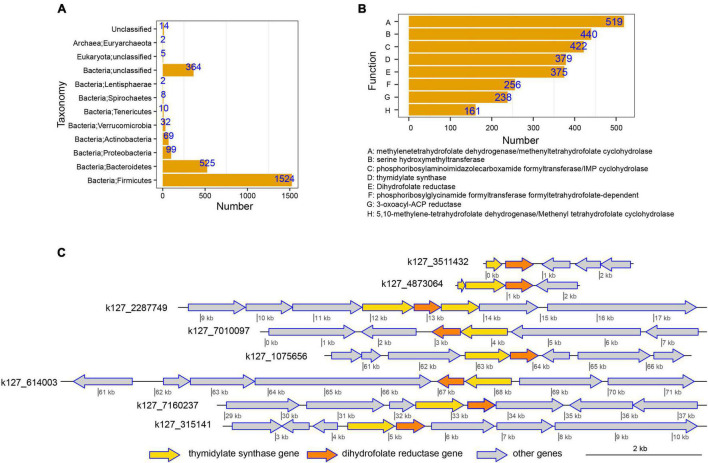
Host-tracking, functional annotation, and physical distribution of genes involved in methotrexate (MTX) metabolism were explored to obtain an in-depth understanding of the interactions between drug response and gut microbiota. **(A)** Taxonomical distribution of genes involved in MTX metabolism. The results suggest that *Firmicutes* and *Bacteroidetes* were the dominant hosts of the genes involved in MTX metabolism and had the potential to metabolize MTX in the intestinal tract. **(B)** Distribution of functional traits for genes involved in MTX metabolism. **(C)** Occurrences of the thymidylate synthase gene and dihydrofolate reductase gene in different assembled contigs were used as an example to reveal the complex and diverse distributions of genes involved in MTX metabolism.

Furthermore, to explore the sources of genes involved in MTX metabolism and their functions, host-tracking and functional annotations were conducted. The host-tracking results revealed that the genes were mainly affiliated with *Firmicutes* (1,524 [57.42%]) and *Bacteroidetes* (525 [19.78%]; Figure 4A and Supplementary Table 2). The functional traits of these genes were mainly annotated as methylenetetrahydrofolate dehydrogenase/methenyltetrahydrofolate cyclohydrolase (19.56%) and dihydrofolate reductase (14.13%; Figure 4B) that can metabolize MTX (Rajagopalan et al., 2002). Besides, the distribution of these genes in different contigs was diverse, even for the same genes that have the same function and highly similar sequences. For example, the occurrence of dihydrofolate reductase gene is strong with thymidylate synthase gene, and its distribution in different microbiota (assembled contigs) was diverse and complex (Figure 4C and Supplementary Table 3), which reflected that the potential metabolic capability of different gut microorganisms on MTX metabolism is diverse.

## Discussion

In the past decades, many studies have proven that the gut microbiota plays an essential role in maintaining human health and participates in the occurrence and development of diseases, including inflammatory diseases [e.g., RA Manasson et al. (2020)] and other diseases (Wu and Wu, 2012; Carding et al., 2015). Researchers have proven that the gut microbiome is in a state of homeostasis under normal circumstances, and the alterations of the human gut microbiome can be caused by several factors, such as environmental factors, host genetics, diet, and drugs. In particular, the theory of “Affinal Drug and Diet” of traditional Chinese medicine and the declaration of Hippocrates (“Let medicine be the food and let food be the medicine”) have highlighted the importance of diet and drugs in the composition of the human gut microbiota. In recent years, researchers have focused on the interactions between diseases and gut microbiota to develop effective treatment methods to treat the diseases related to the gut microbiota, such as fecal microbiota transplantation and probiotics (Han et al., 2020b). Several studies have proven that modulating the homeostasis of the gut microbiota can treat joint diseases (Nayak, 2021) and RA (Pan et al., 2019; Fan et al., 2021). In particular, the relationship between drugs and gut microbiota has become another important research hotspot. For example, by integrating the multi-omics datasets of 2,173 European residents from the MetaCardis cohort, a previous study reported the explanatory power of drugs for the variability in host and the gut microbiome that contributed to the disease and provided a new hypothesis for drug–host–microbiome interactions in cardiometabolic diseases (Forslund et al., 2021). Obtaining an in-depth understanding of the relationship between drugs and the gut microbiome, in particular, the mechanism of drug metabolism by gut microorganisms and the alterations in the gut microbiome affected by drugs, would provide a new view on the drug–host–microbiome axis and has potential implications for treatment of diseases (Enright et al., 2016; Walsh et al., 2018; Weersma et al., 2020).

The occurrence and development of RA, as a widely recognized autoimmune disease, are associated with alterations in the gut microbiota (Zhang et al., 2015; Horta-Baas et al., 2017; Brown et al., 2020). However, the pathology of RA remains elusive, and the effect of clinical treatment is still unsatisfactory. For example, up to 50% of patients treated with MTX could not obtain a clinically effective outcome; the possible reason might be associated with the gut microbiota (Artacho et al., 2021), which suggests that the metabolism of drugs is associated with the gut microbiota. To date, despite the development of several effective therapies, the field of rheumatology lacks tools to help clinicians decide on which drugs are most likely to benefit the patients. Identifying such a tool is urgent and essential for the clinical treatment of RA.

Hence, to address this issue, we searched and downloaded the gut metagenome datasets related to RA patients in a public database. Three available datasets were obtained (Zhang et al., 2015; Artacho et al., 2021), and only BioProject PRJNZ682730 recorded the response to MTX, so MTX was selected as the model drug. In addition, to compare the taxonomical and functional compositions of gut microbiota and the potential metabolic capability of gut microbiota in healthy individuals and RA patients, the gut metagenome datasets were also collected from a healthy Chinese cohort (Li et al., 2017). As a result, a total of 533 human gut metagenome datasets were obtained to investigate the relationship among RA, drug response, and gut microbiota and to reveal the underlying mechanism of different effects of the same drug. To obtain additional potential genes/proteins involved in MTX metabolism, we used those proteins obtained from the PDB database as seed sequences and searched in NR database, which significantly increased the number of potential proteins. These proteins were used to construct a database to profile the compositions of the proteins involved in MTX metabolism and the corresponding genes in healthy individuals and RA patients. We found that the genes involved in MTX metabolism were detected in healthy individuals and RA patients (Figures 2A,B), indicating that the universality of genes related to MTX metabolism in the human gut microbial community and gut microbiota harbors the potential metabolic capability on MTX. However, the number of genes involved in MTX metabolism differed among the cohorts, which suggests that the construction of a comprehensive non-redundant protein set should be established in future study. Furthermore, we also calculated the relative abundance of the 2,654 genes in the training and testing data of the BioProject PRJNZ682730. We found that the compositions of these genes in the gut microbial communities significantly differed between the R and NR groups (Figure 2C). This finding suggests that the response of MTX in RA patients could be associated with the gut microbiota, particularly the compositions of genes involved in MTX metabolism. Hence, these results revealed that gut microbiota possibly participates in the metabolism of drugs in the clinical treatment of RA patients. Whether the structure of gut microbial communities, including taxonomical, functional, and gene compositions, can serve as a classifier to predict the effectiveness of a clinical medication should be verified.

Subsequently, we compared the composition of the genes involved in MTX metabolism between the R and NR groups, and our results revealed that the potential metabolic capability of gut microbiota on MTX metabolism has a significant difference between the gut microbial communities of the R and NR groups. In addition, it should be noted that the potential metabolic capability of gut microbiota on MTX metabolism was evaluated based on the abundance of the genes; as such, the results might be not consistent with the real abundance of proteins involved in MTX metabolism. In this regard, transcriptome and protein sequencing are optional in future studies. Moreover, taxonomical, functional, and gene biomarkers were identified with LEfSe (Figures 3A,B). Interestingly, *P. copri*, belonging to the family Prevotellaceae, has been proven to be significantly altered in individuals with preclinical RA stages compared with the healthy controls (Drago, 2019), which was identified as a fecal biomarker for RA diagnosis (Alpizar-Rodriguez et al., 2019) and a taxonomical biomarker for the R and NR groups. Furthermore, to better serve the clinical medication, we established models based on clinical information, as well as taxonomical, functional, and gene biomarkers by using the random forest algorithm and then verified them in the testing data of BioProject PRJNA682730 (Figure 3D). Our results showed that the accuracy of the models reached 80.95%, with the AUC value of 0.8333, which is approximately equal to the value reported earlier (AUC = 0.84, models built with different features) (Artacho et al., 2021), suggesting that the proposed model can guide clinical application.

In addition, to better understand the sources of genes involved in the MTX metabolism, we conducted the host-tracking analysis and explored the distribution of the host taxonomy of these genes. Our results showed that the taxonomies of these genes are mainly classified into Firmicutes and Bacteroidetes (Figure 4A). Previous studies have demonstrated that the ratio of Firmicutes to Bacteroidetes is a biomarker that represents the dysbiosis of the gut microbiota (Grigor’eva, 2020), and Firmicutes and Bacteroidetes play an important role in the metabolism of substrates. For example, foods rich in fibers are mainly degraded by Firmicutes and Bacteroidetes into short-chain fatty acid such as butyrate, which could have a positive effect on gut dysbiosis by reducing intestinal permeability, promoting bacterial translocation, and limiting inflammation (De Santis et al., 2015; Mitsou et al., 2017). In particular, in terms of the pathogenic factors of RA, smoking affects the relative abundance of Firmicutes and Bacteroidetes (Savin et al., 2018), which suggests that the taxonomical members of these two phyla play a key role in the development and clinical treatment of RA. These results remind us that the gut microbiota plays an essential role in the metabolism of substrates, including drugs. Nevertheless, it should be noted that the taxonomical classification of each gene cannot be identified at the species level, which is a challenge to gain insight into which bacteria harbor the metabolic ability of MTX.

Overall, our results demonstrate that the response mechanism to MTX in RA patients is highly probably related to the catabolic ability of the drug in the gut microbiota and suggest that the profile of potential metabolic capability is an important feature for determining whether the host responds to MTX. Our findings provide a solid foundation for addressing individual differences in drug responses in RA or even other diseases and could elicit effects on clinical management not only for RA but also other gut microbiome–related diseases in the following contexts: First, it suggests that the profile of the potential genes involved in drug metabolism is an important feature for affecting the efficiency of drugs, and clinical treatment strategy should incorporate the compositions of these genes of the gut microbial communities. Second, a suitable model could be constructed by combining gene profiles and other valuable information in the gut microbiome to determine whether the host responds to drugs before clinical treatment (Onuora, 2021). Finally, this work provides an idea that other diseases related to gut microbiota can be studied by similar methods to determine the clinical role of various drugs.

## Conclusion

The gut microbiota plays an essential role in the occurrence and development of diseases, such as inflammatory disease, including RA. The interactions between the gut microbiota and clinical treatment have become a research hotspot. However, the mechanism underlying the effect of microbiota on drug efficiency remains unclear, and a decision model should be established to guide the selection of drugs during the clinical treatment of diseases. In this study, we collected the gut metagenome datasets related to RA patients treated with MTX from a public database, as well as the metagenome datasets of healthy individuals and other RA patients. A total of 533 human gut metagenome datasets were collected from four cohorts. We profiled the taxonomical and functional compositions and identified the genes involved in MTX metabolism between the R and NR groups. Our results showed that the composition of genes involved in MTX metabolism significantly differed between the R and NR groups. These genes were mainly affiliated with Firmicutes and Bacteroidetes. Based on the taxonomical, functional, and gene biomarkers, a random forest model with high accuracy was constructed to provide a decision model for the clinical application of drugs. In addition, the constructed databases can be available at the website: https://github.com/LabHanmz/Drug_response_in_RA. Overall, our study highlights that the potential metabolic capability of gut microbiota on drug metabolism is associated with the drug response and provides a solid foundation for exploring the interactions among drug metabolism, drug response, and gut microbiota and deepens the understanding of the mechanism of drug metabolism mediated by gut microbiota. Our work provides a basis for the clinical management of diseases related to gut microbiota.

## Data Availability Statement

The original contributions presented in the study are included in the article/Supplementary Material, further inquiries can be directed to the corresponding authors.

## Author Contributions

MH, ZC, and SH designed the study. MH, NZ, and YM downloaded the datasets and conducted the data analysis. MH, ZC, NZ, YM, BH, MR, and SH wrote the initial draft of the manuscript. All authors read, modified, and approved the final manuscript.

## Conflict of Interest

The authors declare that the research was conducted in the absence of any commercial or financial relationships that could be construed as a potential conflict of interest.

## Publisher’s Note

All claims expressed in this article are solely those of the authors and do not necessarily represent those of their affiliated organizations, or those of the publisher, the editors and the reviewers. Any product that may be evaluated in this article, or claim that may be made by its manufacturer, is not guaranteed or endorsed by the publisher.

## Footnotes

1
https://github.com/LabHanmz/Drug_response_in_RA

2
https://github.com/dutilh/CAT
